# Curcumin in Depression: Potential Mechanisms of Action and Current Evidence—A Narrative Review

**DOI:** 10.3389/fpsyt.2020.572533

**Published:** 2020-11-27

**Authors:** Tahiana Ramaholimihaso, Fayçal Bouazzaoui, Arthur Kaladjian

**Affiliations:** Psychiatry Department, University Hospital, Reims, France

**Keywords:** curcumin, turmeric, depression, mechanism of action, neuroprotective agent, inflammation, NLRP3, dietary supplement(s)

## Abstract

Major depressive disorder (MDD) is one of the most prevalent and debilitating disorders. Current available treatments are somehow limited, so alternative therapeutic approaches targeting different biological pathways are being investigated to improve treatment outcomes. Curcumin is the main active component in the spice turmeric that has been used for centuries in Ayurvedic medicine to treat a variety of conditions, including anxiety and depressive disorders. In the past decades, curcumin has drawn researchers' attention and displays a broad range of properties that seem relevant to depression pathophysiology. In this review, we break down the potential mechanisms of action of curcumin with emphasis on the diverse systems that can be disrupted in MDD. Curcumin has displayed, in a number of studies, a potency in modulating neurotransmitter concentrations, inflammatory pathways, excitotoxicity, neuroplasticity, hypothalamic–pituitary–adrenal disturbances, insulin resistance, oxidative and nitrosative stress, and endocannabinoid system, all of which can be involved in MDD pathophysiology. To date, a handful of clinical trials have been published and suggest a benefit of curcumin in MDD. With evidence that is progressively growing, curcumin appears as a promising alternative option in the management of MDD.

## Introduction

Major depressive disorder (MDD) is the most common psychiatric disorder (1). In 2017, the World Health Organization announced that indeed depression was the leading cause of disability and ill health worldwide, with more than 300 million people living with depression (2).

Major depressive disorder is characterized, by the 5th Edition of the Diagnostic and Statistical Manual of Mental Disorders (DSM-5), by a depressed mood, loss of energy, a markedly diminished interest or pleasure, psychomotor retardation (or agitation), feelings of worthlessness or excessive or inappropriate guilt, insomnia or hypersomnia, significant weight loss, diminished ability to concentrate, and recurrent thoughts of death.

The MDD pathophysiology is recognized as being complex, ranging from genetic predispositions to interactions between environmental factors, and multiple biological systems. Among those, we can name disruption in the monoamine systems, which has been the prime hypothesis in MDD pathophysiology, as well as neurodegenerative processes, notably through hypothalamic–pituitary–adrenal (HPA) changes or inflammatory processes (3).

The management of MDD has long been based on psychological interventions, and although guidelines presently suggest to practitioners to combine psychological interventions and pharmacological treatments, the discovery of antidepressant medications has made them a staple in MDD management. However, as decades have gone by, the development of antidepressants somehow reached a plateau. Although they proved to be effective (4), they are still not optimal as we can see remission rates of around 30% after a first-line selective serotonin reuptake inhibitor therapy (5) and a cumulative remission rate of only 70% after a fourth line of pharmacological treatment (6). Moreover, lack of adherence to antidepressant medications is frequently observed, the presence of side effects being a major cause of this non-compliance.

The problem of treatment resistance has underscored the need for new management strategies, and as concepts and views on MDD pathophysiology have evolved, so have therapeutic alternatives. For this reason, we could see a growth of neurostimulation in the past decades (7) as well as the role of physical activity in the management of MDD (8, 9). Consequently, interest in complementary and alternative medicine has been growing.

As for alternative medicines, anti-inflammatory, antioxidants, and neuroprotective compounds thought to counteract the degenerative processes frequently associated with MDD have been a new concern of exploration for treatment or adjuvant therapy. Notably, the Canadian Network for Mood and Anxiety Treatments clinical guidelines recently inserted some alternative medicines such as omega 3, acetyl L carnitine, Lavandula, or saffron among adjunctive treatments for the management of MDD in adults (9).

Turmeric (*Curcuma longa*) is a yellow spice, part of the ginger family (*Zingiberacear*). It has been empirically used for centuries in Ayurvedic and traditional Chinese medicine in a wide variety of diseases and conditions (10). Research conducted in the last half century has revealed that the active compounds of turmeric were curcuminoids, which are polyphenolic pigments that give turmeric its yellowish color. Curcumin is the primary curcuminoid and main active component in turmeric and the compound for which most studies have been done.

In the past decades, there has been a surge of interest in curcumin as evidence about its efficacy in a wide variety of diseases is growing, including cardiovascular, autoimmune, and neurodegenerative diseases as well as diabetes and cancers (11, 12).

Thus, curcumin displays a broad range of properties that are relevant in the pathophysiology of depression. It has been demonstrated to possess an antidepressant activity in various animal models as well as in clinical trials. A dozen randomized controlled clinical trials have indeed been conducted (13), altogether suggesting that curcumin may be effective as a treatment (or adjunct treatment) of MDD *via* multiple mechanisms of action.

We will discuss in this review the potential mechanisms of action underlying the efficacy of curcumin in depression, giving us an overview of the current concepts in the pathophysiology of depression, and we will also discuss the existing evidence of the efficacy of curcumin in the treatment of depression.

## Curcumin and Neurotransmitters

### Curcumin and Monoamines

The monoamine deficiency theory has been the primal causative model of major depression and has accompanied the rise of antidepressants. The current pharmacologic arsenal is based on this theory as the main antidepressants, such as selective serotonin reuptake inhibitors, serotonin–noradrenaline reuptake inhibitors, and monoamine oxidase (MAO) inhibitors, are designed to increase the availability of monoamines (serotonin, noradrenaline, dopamine). According to this theory, it is thought that the depletion of these neurotransmitters in the central nervous system (CNS) constitutes the core of depression pathophysiology (14).

Evidence of curcumin being able to influence levels of monoamines in the central nervous system has emerged from animal and *in vitro* studies conducted over the past two decades. In a study, Bhutani et al. (15) showed that curcumin reversed the depressive-like behavior induced by chronic stress on mice and enhanced the serotoninergic and dopaminergic transmission alongside an inhibition of the MAO-A. Kulkarni et al. (16) had similar results in a study on rats in which curcumin dose-dependently increased serotonin and dopamine as well as inhibited the monoamine oxidase enzymes. More recent studies also showed that curcumin could elevate norepinephrine, serotonin, and dopamine in the frontal cortex, hippocampus, and striatum in rats (17–22).

Wang et al. (23), in an animal study on the effects of curcumin on serotonin (5-HT) receptors, stated that the antidepressant-like effect that they could notice was related to the serotoninergic system, possibly due to an interaction with 5-HT1A/1B and 5-HT2C receptors. Similarly, Xu et al. (24) conducted a study in which the antidepressant effect of curcumin seemed related to the expression of 5-HT1A receptors, as 5-HT1A receptor mRNA levels across all hippocampal subfields were increased after curcumin administration. These results have been replicated by more recent studies like by Li et al. (25) or Lian et al. (26) in which they noticed an upregulation of 5-HT1A receptor expression after curcumin administration in chronically stressed mice and a prevention of its antidepressant-like effect with the administration of a 5-HT1B receptor antagonist.

Taken together, these animal studies strongly support the hypothesis that curcumin can modulate monoaminergic systems in pre-clinical rodent models.

### Curcumin and Glutamate

In 1959, Crane (27) made an observation that the anti-tuberculosis agent d-cycloserine, which acts as a glutamatergic modulator, could possess an antidepressant effect, yet this observation gained little attention during the following decades until 2000, when Berman *et al*. (28) reported that ketamine, an antiglutamatergic anesthetic agent, could induce a rapid antidepressant effect in severely depressed patients. This triggered vigorous research to understand how modulating glutamate signaling could be beneficial in depression and to develop new antidepressants targeting glutamate neurotransmission. It is worth noting that ketamine has now proven its efficacy in treating depression (29).

Glutamate is the major excitatory neurotransmitter in the central nervous system and has a vital role in the regulation of synaptic plasticity. By binding to its receptors, notably NMDA receptors (NMDAR), glutamate will modulate post-synaptic plasticity as well as exert longer-term changes in synaptic strength and neuroplasticity. However, the abnormal elevation of NMDAR signaling leads to deleterious effects on neurons (30). As such, excitotoxicity is the phenomenon associated with excessive glutamate release and subsequent overactivation of NMDA receptors and has long been described (31).

A number of reports suggest that the glutamate system and excitotoxicity are involved in MDD pathophysiology. For example, glutamate levels have been shown to be elevated in the plasma, cerebrospinal fluid, and brains of patients with depression (32).

Chronic stress is believed to lead to detrimental changes within glutamate synapses, including reduced extracellular glutamate clearance by glia, especially in the pre-frontal cortex, leading to increased extrasynaptic glutamate levels and excitotoxicity, potentially contributing to synaptic loss (33). Hence, in a study conducted by Lin et al. (34), curcumin was shown to inhibit the liberation of glutamate in the rat pre-frontal cortex, counteracting this phenomenon in a similar way (yet greater) to that of fluoxetine (suggesting that “classic” antidepressants like fluoxetine also act on the glutamate pathway).

A study by Zhang et al. (35) suggested that the antiglutamatergic action of curcumin could be mediated by the GluN2B subunit of NMDA receptors. Also, they noted that the administration of a sub-effective (that did not produce antidepressant effect when administered alone) dose of curcumin produced an antidepressant-like effect when paired with a sub-effective dose of fluoxetine, leading to the hypothesis of a synergistic interaction between NMDA and 5-HT receptors. These results have been clarified by further *in vitro* studies showing that curcumin could reverse glutamate-induced neurotoxicity on hippocampal cells and downregulate the expression of the GluN2B subunit of NMDA receptors (36, 37). The antagonization of the GluN2B subunit seems to have a crucial role in the effect of anti-NMDAR drugs such as ketamine, as the activation of this subunit inhibits the synthesis of certain synaptic proteins, such as brain-derived neurotrophic factor (BDNF), thus altering synaptic function (38). Hence, the antagonization of GluN2B subunit permits an enhancement of synaptic function. For instance, memantine, which is also an antagonist of NMDA receptors, after acute administration, had no effect on hippocampal BDNF and failed to show immediate antidepressant behavioral effects in an animal depression model (39). However, the prolonged administration of memantine was associated with an increase in BDNF levels and hippocampal cell proliferation while displaying antidepressant-like effects in other animal studies (40, 41). In studies displaying the action of curcumin on the glutamatergic system, these effects were associated with increased levels of BDNF (23, 42) that might account for its antidepressant efficacy.

Collectively, those results suggest that an inhibitory effect of curcumin on excitotoxicity may be one of the mechanisms underlying its antidepressant effects.

## Depression, Inflammation, and Curcumin

In the past decades, as the monoamine depletion theory has been the prime model of depression pathophysiology, other hypotheses have emerged. One of them implies that inflammation has a key role in depression pathophysiology. This hypothesis was prompted by the comparison we can make between “sickness behavior” and depression symptoms like anorexia, reduction of locomotor activity, anhedonia, and cognitive disturbances (43) which can be found in both conditions, and some studies showed that these types of symptoms in depression were positively correlated with C-reactive protein levels (CRP) (44, 45). Furthermore, some reports pointed the elevation of inflammatory cytokines [mainly interleukin-1 (IL-1), interleukin-6 (IL-6), and tumor necrosis factor-α (TNF-α)] in depression (46–48). A meta-analysis displaying a reduction of inflammatory cytokine levels after antidepressant medication administration brought further support for the relationship between depression and inflammation (49). There have also been studies showing that cytokines or CRP levels could predict the antidepressant effect of the therapeutics used, including antidepressant medication (50, 51), physical exercise (52), or electroconvulsive therapy (53). While the meta-analysis performed did not show increased inflammation in every patient, its presence may be relevant to a subset of patients (45, 54), especially since it can guide the therapeutics.

### Curcumin and Immunoinflammatory Pathways

As stated in the introduction, most of the first studies trying to unravel the beneficial effects of curcumin came from research on cancer and inflammatory diseases, and many anti-inflammatory effects of curcumin are now being acknowledged (55). A number of studies have indeed shown that curcumin could inhibit TNF production by macrophages and downregulate its expression by modulation of its transcription factors. Notably, it has been shown that curcumin was able to down-modulate the activation of nuclear factor kappa beta (NF-κβ) (56).

A study conducted by Wang et al. (57) in 2014 has reported the effects of curcumin on inflammation and depressive symptomatology in mice treated with lipopolysaccharide (LPS). This study showed that administrating curcumin reverted the depressive-like behavior and attenuated LPS-induced microglial activation and overproduction of pro-inflammatory cytokine (interleukin-1β and tumor necrosis factor-α). It could also inhibit LPS-induced NF-κβ activation in the hippocampus and pre-frontal cortex (PFC). In addition, curcumin could counteract the increase in the levels of nitric oxide synthase and cyclooxygenase-2 mRNA in the hippocampus and pre-frontal cortex. Jangra et al. (58) reported similar results, as the administration of curcumin in LPS-treated mice improved the depressive-like symptoms, reversed the depletion of glutathione level in the hippocampus, and decreased the level of pro-inflammatory cytokines (IL-1β and TNF-α) in the hippocampus.

Fan et al. (59) showed that chronic unpredictable mild stress exposure in mice induced microglia activation and overexpression of the cytokines IL-1β, IL-6, and TNF-α within the medial pre-frontal cortex, effects which were paralleled with neuronal structural changes. They showed that curcumin administration produced antidepressant-like actions and reversed the inflammatory responses and neuronal structural abnormalities, thus concluding that inhibiting the IL-1β pathway could account for curcumin efficacy.

With regards to studies of human patients, a clinical trial conducted by Yu et al. (60) showed that curcumin decreased the inflammatory cytokines IL-1β and TNF-α in depressed patients in comparison with the placebo group. Moreover, it has been shown in other clinical trials that curcumin can lower TNF-α, IL-6, and CRP levels in patients' plasma (56, 61, 62).

All in all, what we can draw from these studies is that curcumin permits a decrease in inflammation, acting on pro-inflammatory cytokine pathways, notably TNF-α, IL-6, and IL-1β in the hippocampus and pre-frontal cortex.

### Curcumin and Inflammasome Activation

Clinical depression is characterized by inflammatory cascades as indicated by elevated concentrations of pro-inflammatory cytokines in the serum and in the CNS tissue. Given the known action of curcumin on some immunoinflammatory pathways, the pivotal role of IL-1β and microglia activation in the pathophysiology of depression, and the relevance of these processes to the therapeutic properties of curcumin, we can highlight a probable role for the inflammasomes.

Inflammasomes are crucial components of the innate immune response that initiate immunological reactions against microbial infections, tissue injury, and other aggressions. The key inflammasome in depression is the NLRP3 inflammasome complex, which, upon activation, consists of a NOD-like receptor protein containing pyrin domain (NLRP3) associated with an adaptor protein ASC and an effector caspase-1 (63).

NLRP3 inflammasome activation is related to microglial activation. Upon initially recognizing an environmental stressor, the microglia can indeed enter in an active state and are thereafter more prone to induce a prolonged inflammatory response following the upcoming exposure to those stressors. NLRP3 inflammasome has drawn interest as an explanatory element as to why primed microglia cells display sensitivity to environmental stressors. In fact, NLRP3 inflammasome acts as a transducer of neuroinflammatory responses. A lowered threshold for NLRP3 activation induces an increased production of inflammatory cytokines, such as IL-1β and IL-18, and then causes a persistent neuroinflammation (64). This chronic inflammation results from chronic exposure to noxious threats that negatively affect the homeostatic feedback mechanisms. In sum, NLRP3 inflammasome is a molecular mechanism that translates psychological stressful stimuli into inflammatory responses.

The activation of the NLRP3 inflammasome relies on a two-step mechanism. It requires a priming signal to initiate the transcription of the NLRP3 inflammasome to above a certain threshold, while a second signal will then promote the formation of the NLRP3 inflammasome by recruiting ASC and pro-caspase-1 proteins.

The priming of NLRP3 transcription is mediated by NF-κβ activation, which can result from the stimulation of membrane-bound receptors by TNF or pathogen-associated molecular patterns like LPS (63) or damage-associated molecular patterns whose presence indicates cellular or metabolic stress. It is worth noting that there were some reports indicating that corticosterone may also stimulate the transcription of NLRP3 *via* glucocorticoid receptor activation, involving the NF-κβ pathway but also independently of NF-κβ activation (65, 66).

Another way of activating NLRP3 inflammasome is the reduction of intracellular potassium (K+) levels *via* the activation of the P2X7 purinergic receptors. Extracellular ATP is released by neurons and astrocytes following excitotoxicity. Activation of these receptors causes potassium efflux in microglia, which is crucial for the activation of the NLRP3 inflammasome (67). Besides that, P2X7 receptor is being investigated as a potential drug target in mood disorders (68). Reactive oxygen species (ROS) can also promote NLRP3 activation (69). An activated NLRP3 inflammasome will then cleave pro-IL-1β and pro-IL-18 into active IL-1β and IL-18.

There have been a few studies highlighting the role of NLRP3 inflammasome in depression. Animal studies investigating this theory showed that chronic stress could stimulate NLRP3 activation in rodent brains and that, accordingly, in the absence of the NLRP3 inflammasome activation, stress did not produce a depressive behavior, anhedonia, or social impairment in those mice (70–73). It has also been shown that NLRP3 is activated in mononuclear cells in patients with depressive disorder and that some antidepressants compounds seem to show a decrease in NLRP3 activation (74).

Concerning curcumin, in an *in vitro* study conducted by Li et al. (75) on rat hippocampal cells, excessive glutamate release induced IL-1β secretion and generation of ROS. Curcumin was able to inhibit the generation of ROS and NLRP3 inflammasome activation assessed by NLRP3 and caspase-1 expression, which resulted in a reduction of IL-1β secretion. In addition, curcumin could prevent glutamate-induced cell apoptosis. In the same vein, Fan et al. (76) showed that curcumin administration could prevent IL-1β-induced apoptosis in stressed rats in the ventromedial pre-frontal cortex and alleviate depressive behavior.

In a study conducted by Zhang et al. (77), rats were exposed to chronic unpredictable stress, and this exposure led to depressive-like behaviors. Curcumin successfully corrected the depressive-like behaviors in those stressed rats. Additionally, curcumin could effectively decrease the mRNA expression of proinflammatory cytokines (IL-1β, IL-6, and TNF-α) and suppress NF-κβ activation as well as inhibit the expression of P2X7 receptors, thus preventing NLRP3 activation. These results were in accord with a study conducted by Wang et al. (78), in which curcumin could inhibit the activation of the P2X7 receptor and thus inhibit the inflammatory response and the microglial activation, thus preventing NLRP3 activation.

It is also worth quoting a recent study conducted by Ozkartal et al. (73) that highlighted the role of nitric oxide synthase (NOS) in the activation of NLRP3 inflammasome; in this study, the administration of NOS inhibitors in chronically stressed mice prevented the activation of NLRP3 inflammasome and the production of IL-1β.

In some reports, depression has been associated with an excessive activity of nitric oxide synthase (79). Also, some antidepressant medications in current use, such as paroxetine, inhibit NOS activity (80). In some other studies, suicidal and depressed patients had elevated levels of plasma NO and its metabolites (81, 82). The overactivation of NOS pathway and subsequently increased nitric oxide has been described as an important pathophysiological factor in neuroinflammation and neurotoxicity processes involved in stress and depression (79, 83) as well as an important modulating factor in the production of neurotransmitters such as noradrenaline, serotonin, and dopamine (79), thus explaining why NOS inhibitors can exert antidepressant and anxiolytic-like effects (73, 84).

In human cultured neurons exposed to quinolinic acid, quinolinic acid increased NOS activity and consequently increased the nitrite levels. In those neurons, curcumin could counteract this increase of NOS activity (85). Studies investigating the influence of curcumin on NOS pathway in animal models have shown that, in rat (86) or pig (87) stress models, curcumin could inhibit NOS hyperactivation and the subsequent increase in hippocampal NO. As previously cited in the study conducted by Wang et al. (57) on mice with LPS-induced depressive symptomatology, curcumin could attenuate the LPS-induced microglial activation and NF-κβ activation in the hippocampus and PFC as well as decreased the levels of NOS in the hippocampus and PFC.

These data suggest that NLRP3 activation can play a major role in inflammatory-related depressive symptoms and that its inhibition by curcumin might be a key feature of its effectiveness.

### Curcumin and Kynurenine Pathway

Indoleamine 2,3-dioxygenase (IDO) is an enzyme that is strongly activated by proinflammatory cytokines (such as TNF-α, IL-1, and IL-6) (88). This enzyme has an important role in depression by catabolizing tryptophan (the primary precursor of serotonin) into kynurenine pathway metabolites: IDO leads to tryptophan depletion and thus inhibits serotonin synthesis. As stated before, the role of serotonin in depression is already admitted. However, research shows that kynurenine pathway metabolites can impact several mechanisms associated with depression, such as pro-apoptotic changes in the CNS or generation of free radicals (89). Preclinical studies have shown that IDO activation results in depressive-like symptoms and that IDO inhibitors could relieve depression-like behaviors in mice (90).

There are two main kynurenine pathway metabolites studied in depression: one of them is quinolinic acid, an NMDA agonist, that is thought to be excitotoxic, thus leading to synaptic loss. It can also disrupt the oxidant/antioxidant balance by increasing the generation of free radicals and induce lipid peroxidation (88). Excess quinolinic acid levels were found in depression (91), however inconsistently (92). The levels of quinolinic acid were still shown to predict the response to antidepressant medications such as ketamine (93). The second main kynurenine pathway metabolite is kynurenic acid, considered to be neuroprotective. Its balance with quinolinic acid is considered to be protective against the excitotoxicity of the latter (94). Moreover, studies reported lower levels of kynurenic acid in depression (92).

*In vitro* studies demonstrated that curcumin could counteract the inflammation-induced overexpression of IDO (95), and more recently, a study conducted by Zhang et al. (77) showed that curcumin could inhibit the stress-induced overexpression of IDO in rats and normalize the quinolinic acid/tryptophane ratio.

Another important co-factor of the increase of IDO is cyclo-oxygenase 2 (COX-2) (96). COX-2 is an enzyme responsible for the production of prostaglandin E2 (PGE2), which is a pro-inflammatory chemical messenger. Studies have shown an increase in PGE2 production and COX-2 expression in depressed patients (97, 98). The peripheral blood cells of patients with recurrent depression also exhibited an increased expression of the genes encoding for COX-2 (99). A number of clinical trials have yielded promising results for the use of COX-2 inhibitors as an augmentation of antidepressant treatments (100). Their use could also help decrease inflammatory cytokine levels (101).

Regarding curcumin, studies have shown that it can downregulate COX-2 expression and PGE2 synthesis *in vitro* (102) and in animal models of depression (103), highlighting its potential role as an alternative natural COX-2 inhibitor option. It is relevant to mention that COX-2 can induce NOS activity and *vice versa* (104). The 2014 Wang et al. (57) study mentioned before (see the previous section) illustrates the interplay between NOS, IDO, and inflammatory cytokines.

Considering the possible role of COX-2 in depression and its role in IDO synthesis and the role of IDO in depression that we illustrated, these data suggest interesting mechanisms of action for the use of curcumin as an augmentation therapy.

### Curcumin and Intestine Hyperpermeability

As the largest mucosal surface in the human body, the intestinal epithelium acts as an interface with the external environment (105). In some situations, the permeability of the intestinal epithelium can be altered in a state also called “leaky gut.”

Lipopolysaccharides (LPS) are large molecules found in the outer membrane of gram-negative bacteria. In a state of “leaky gut,” there will be an increased translocation of those gram-negative bacteria from the intestine into the systemic circulation and, with them, the LPS (106, 107). LPS can then stimulate toll-like receptors, generating an inflammatory process. This will result in pro-inflammatory cytokine secretion and neuroinflammation (108) and also activation of IDO with the previously described consequences (109–111).

Some studies have reported increased serum IgM and IgA levels directed against the LPS of gram-negative enterobacteria in depressed patients, reflecting an increased translocation of LPS from those gram-negative enterobacteria, with the authors of such studies concluding that depressive disorder was accompanied with intestinal hyperpermeability (107, 112).

In animals, LPS induces depressive behaviors (110). Thus, a number of studies investigating the anti-inflammatory effects of curcumin were carried out using LPS models. In such models, curcumin reversed LPS-induced behavioral and molecular changes. Moreover, a handful of *in vitro* studies have shown that curcumin could increase the expression of tight junction proteins, thus preventing the disruption of tight junction organization and decreasing LPS increase due to paracellular permeability (113).

These data suggest that one antidepressant mechanism of action of curcumin could be its ability to counteract this state of gut permeability and subsequent LPS-induced inflammation.

## Depression, Metabolism, and Curcumin

### Curcumin and HPA Axis

The HPA axis is a central system in the body's stress response, and abnormalities in its activity have long been noted in MDD (114). Studies have shown that depression was associated with impairment in the responsiveness to glucocorticoids and a subsequent hypersecretion of CRH. This phenomenon is known as glucocorticoid resistance and can, in turn, prime the inflammatory response (114). We have indeed highlighted the role of corticosterone in the activation of NLRP3 inflammasome (65, 66). Depression is also associated with an increased size and activity of the pituitary and the adrenal glands (115).

Based on these assumptions, animal models of depression related to stress and elevated cortisol levels have been developed. In such models, curcumin has been shown to alleviate the depressive symptoms and other physiological alterations induced by cortisol.

Li et al. (25) showed that, in stressed rats, curcumin could restore the normal level of corticosterone. Rinwa and Kumar later found similar results (116). Researchers showed that curcumin could protect against corticosterone-induced neurotoxicity and downregulation of mRNA levels of serotoninergic receptors in a rodent model (5-HT1A, 5-HT2A, and 5-HT4) (117). Curcumin could also normalize the adrenal gland size in stressed rats (118). Furthermore, curcumin decreased the salivary cortisol concentrations in depressed patients compared with the placebo group in the clinical trial of Yu et al. (60).

### Depression, Insulin Sensitivity, and Curcumin

Insulin receptors are present throughout the brain, and they play a central role in regulating its use of glucose for energy (119). Moreover, some of insulin's actions are specific for the CNS, like promoting neuronal survival or synaptic plasticity and regulation of brain functions including memory, cognition, learning as well as attention (120, 121). Insulin also inhibits norepinephrine and serotonin reuptake and downregulates alpha-2 receptor expression in hypothalamic neurons (122).

Consequently, there has been a hypothesis suggesting that insulin resistance played a role in depressive disorder pathophysiology, which was reinforced by epidemiological studies. For example, adults with insulin resistance are more at risk for the development of future depression (123), and a meta-analysis has shown an association between insulin resistance and depression incidence (124). Significantly, insulin resistance interacts with other depressogenic processes. Given the insulin functions in the brain, it has also been shown that reduced brain sensitivity to insulin can manifest as impaired neuroplasticity and disturbances in neurotransmitter's release and uptake (125). Insulin resistance has also been shown to be associated with altered dopamine signaling in rodents (126).

Inflammation and oxidative stress are both involved in the pathogenesis of insulin resistance (127, 128). In turn, insulin has antioxidant properties which become disrupted following insulin resistance (129). As described earlier, oxidative stress resulting from the disruption of insulin functions can, in turn, trigger the production of pro-inflammatory cytokines. The desensitization of glucocorticoid receptors that can be associated with insulin resistance (130) may potentiate this depressogenic cascade.

There is an emerging research field trying to reposition some antidiabetic medications as new means in depression management like GLP1 functional agonists, pioglitazone, or even metformin (131).

Concerning curcumin, Shen et al. (132) have conducted a study on mice with unpredictable chronic mild stress in which they showed that curcumin could attenuate insulin resistance alongside a decrease in depressive-like symptoms. There were also other studies showing the positive effects of curcumin on insulin resistance in *in vitro*, animal, or even human studies (133).

## Depression and Neuroplasticity, Curcumin, and Neuroprotection

The 1990s witnessed the rise of the “neurotrophic” hypothesis of depression, which associated chronic stress and depression with a deficit in BDNF and demonstrated that traditional antidepressants increased BDNF expression (134). Hence, by the early 2000s, studies progressively exhibited increasing evidence of the presence of cellular atrophy and neuronal death in major depressive disorder. Studies also showed increased growth factor (such as BDNF) levels associated with antidepressant therapies such as physical exercise (135), antidepressants (136), electroconvulsive therapy (137), or lithium (138). This supports the hypothesis that the neurotrophic effects of antidepressants account for their efficacy in treating MDD, with some authors even viewing BDNF as an essential determinant of antidepressant efficacy (136).

We have already underlined the neuroprotective effect that curcumin could exert *via* its inhibition of inflammatory pathways and the mitigation of glutamate excitotoxicity (59, 76). This is particularly illustrated by a study conducted by Choi et al. (103) in which curcumin administration permitted an improvement of the depressive behavior induced by chronic unpredictable stress in mice alongside reduced hippocampal neuronal cell death, attenuated long-term depression, increase in BDNF, and COX-2 inhibition, suggesting neuroprotection *via* anti-inflammatory effects. Curcumin can also exert neuroprotective effects by stimulating the production of neurotrophic factors, especially BDNF.

The extracellular signal-regulated kinase (ERK) is a subfamily of mitogen-activated protein kinase (MAPK), an essential family of serine/threonine kinase regulating cellular growth, differentiation, and survival in proliferative cells including brain cells (139). ERK, alongside BDNF, has shown to be downregulated in the PFC and hippocampus of depressed humans and animals, and antidepressants could, in turn, reverse the hypoactivity of ERK and alleviate depression-like behaviors. Reciprocally, in animal studies, blocking the BDNF pathway resulted in a loss of effects of antidepressants (140).

The transcription factor CREB is also a downstream target of ERK, and other studies found that CREB phosphorylation was decreased in the frontal cortex and the hippocampus of stressed humans and animals, accompanied with a decrease in ERK activity. Antidepressants reversed the reduction of CREB phosphorylation in stressed animals (140). In short, the activation of the ERK pathway by antidepressants increases the expression of nuclear CREB, which facilitates the expression of neurotrophic/neuroprotective proteins such as the BDNF.

In an adult male Wistar–Kyoto rat model of depression, Hurley et al. (141) showed that curcumin had an antidepressant activity and found an increase in hippocampal BDNF. Similarly, Xu et al. (24) showed that administrating curcumin to chronically stressed mice increased the hippocampal BDNF. Liu et al. (142) showed similar results in chronically stressed mice with a positive effect on stress-induced learning and memory deficits and an upregulation of BDNF and ERK in the hippocampus.

Some authors have also shown a neuroprotective effect of curcumin in the amygdala. For example, Zhang et al. (143) showed in a study that curcumin could increase BDNF levels in rat amygdala *via* ERK phosphorylation and alleviate depressive behavior. They later replicated those results (144). Similarly, Abd–Rabo (145) could show that the administration of curcumin in ovariectomized rats could alleviate depressive behavior and upregulate the BDNF mRNA in the limbic system. With regards to human studies, Yu et al. (60) showed, in a clinical trial, that curcumin increased the plasma BDNF levels compared to the placebo group.

These studies suggest that curcumin can alleviate depressive behavior through activation of the ERK/BDNF neurotrophic pathway, especially in the hippocampus, the pre-frontal cortex, or the amygdala that are involved in depression pathophysiology.

## Oxidative Stress, Depression, and Curcumin

As stated in the introduction, depression pathophysiology may involve several interconnected biochemical pathways including nitrosative stress and oxidative stress. Generally, oxidative stress is defined as an imbalance between the production of reactive oxygen and nitrogen species as well as the efficiency of enzymatic (like catalase, glutathione peroxidase, and superoxide dismutase) and non-enzymatic (like reduced glutathione and uric acid) antioxidative systems, so cells are said to be in a state of oxidative stress when the level of reactive oxygen species exceeds the endogenous antioxidant defense mechanisms (for e.g., the enzymes catalase, glutathione peroxidase, or superoxide dismutase) (146).

Studies to date indicate that patients with depression have lower levels of antioxidative systems, and at the same time, they display an increased amount of oxidative stress markers when compared with healthy individuals (147, 148). Some antioxidant therapeutics, like N-acetyl-cysteine, seem to show some effect in depression treatment (149).

Recently, Naqvi et al. (150) have shown that, on chronically stressed mice, curcumin administration could correct depressive behaviors and improve memory functions as well as improve oxidative stress as measured by the peroxidation of lipid and the antioxidant enzyme activities. Fidelis et al. (151) administrated curcumin in a model of oxidative stress induced by beta amyloid infusion in mice and noticed an antidepressant-like effect as well as a reduction of the Aβ-generated oxidative stress in the pre-frontal cortex, as evidenced by the reactive species levels and the superoxide dismutase and catalase activities. Rinwa and Kumar (116) exposed mice to a chronic unpredictable stress that significantly impaired the oxidative parameters (elevated malondialdehyde and nitrite concentrations and decreased glutathione and catalase levels) and mitochondrial enzyme complex activities. Those effects were reversed by the administration of curcumin; there was an improvement in the depressive-like behavior as well. Jangra et al. (58) were also previously able to show that curcumin could reverse glutathione depletion in the hippocampus induced by LPS administration. Concerning nitrogen species and nitrosative stress, we previously addressed this issue and how curcumin could oppose this noxious phenomenon by its action on NOS.

As we have stated before, oxidative stress and mitochondrial dysfunction leading to the formation of reactive oxygen species are involved with other pathophysiological mechanisms such as inflammation. As a consequence, tackling this pathogenic cascade could be an interesting way to improve treatment outcomes in depression.

## Curcumin and the Endocannabinoid System

Two kinds of cannabinoid (CB) receptors have been found in the human body: CB1 and CB2 receptors. They can activate multiple cell signaling pathways to regulate the neurotransmitter release process (152). CB1 receptors are mainly distributed in the central nervous system and may be related to the cannabinoid functions of memory and cognition regulation as well as motor control and show relatively low expression levels in the peripheral nervous system (153). On the other hand, CB2 receptors are mostly distributed in peripheral immune cells, mainly affecting immune regulation (153).

Considering the regulatory function of CB receptors on mood (154) and their distribution in brain regions related to mood and reward (pre-frontal cortex, limbic system, raphe nuclei) (155), some authors proposed that the endocannabinoid signaling pathway may be involved in the formation and development of depression, as suggested by diverse animal studies showing the apparition of depressive behaviors with the blockade of CB1 receptors (156). We could also cite the example of rimonabant (a CB1 antagonist used to treat obesity) that has been forced out of the market due to anxiety and depression reported as frequent and important side effects (157) and studies reporting that the use of rimonabant could inhibit positive emotional memory as well as the reward system (158). Based on these observations, reports have also shown that the activation of CB1 receptors could result in similar behavior and biochemical changes as those caused by antidepressants (159).

He et al. (160) tested an association of curcumin and HU-122, a cannabinoid known for having no effect on CB receptors. They showed some efficacy in a corticosterone-induced model of depression. The administration of this dual drug could prevent corticosterone-induced neural cell apoptosis and improve the dopamine levels, especially in the hippocampus and the striatum. Alongside those effects, the authors noted an enhanced expression of CB1 receptors, which could have an important role in the antidepressant potency of curcumin. Hassanzadeh and Hassanzadeh (161) suggested that the endocannabinoid system could have a pivotal role in emotion regulation and neuroplasticity exerted by curcumin as they showed in their study that chronic curcumin administration in rats could increase endocannabinoid levels as well as nerve growth factor levels in key structures like the amygdala and the hippocampus. Witkin et al. (162) noted that curcumin had no effect on depression-like behavior on CB1 KO (–/–) mice, highlighting the role of CB1 receptors in curcumin efficacy.

In sum, these data highlight the role of CB1 receptors as a novel and multimodal target of curcumin.

## Translation into Clinical Practice

To date, clinical trials have yielded conflicting results regarding the efficacy of curcumin in depression; however, there has been two meta-analyses concluding that curcumin could be effective in depression: the first one was in 2017 (163), which included six studies with a total of 377 patients comparing the use of curcumin to placebo, supporting a significant clinical efficacy in depression, and the second one was conducted by Fusar-Poli et al. (13), in which curcumin was evaluated as an add-on therapeutic, that included 10 studies with a total of 531 patients, supporting the efficacy of curcumin as an add-on.

However, even though there were moderate to large effect sizes in those meta-analysis, it is important to note that not every study yielded significant results and that the studies were heterogeneous, especially with the formulations and the dosage of curcumin used. Yet a concern about curcumin has been its bioavailability, and thus diverse formulas are being developed to enhance its bioavailability, like the use of curcumin nanoparticles, liposomal encapsulation, emulsions, lipid complexes, use of piperine, or development of synthetic curcumin analogs (164, 165). However, no statistical analysis could be performed in those meta-analysis regarding a potential difference in efficacy between formulas due to the small number of studies, so further clinical trials with diverse curcumin formulations will be important since, in animal studies, different magnitudes of effects on the behavioral, molecular, and electrophysiological levels have been reported depending on the curcumin formula administered (20, 151). Nonetheless, the meta-analyses conducted did not show significant differences with respect to the dosage of curcumin used (> or ≤500 mg/day). Moreover, it is important to note that the clinical trials conducted were generally small, with samples varying from 14 to 123 (13). Thus, enlarging samples in future studies would be desirable.

Curcumin generally appears to be well-tolerated, with only mild side effects like yellow stool, headache, or diarrhea noted in doses up to 12 g/day (166).

On another hand, most of the studies were conducted in Asian countries, where dietary regimens usually include curcuminoids, so it would be interesting to test the effects of curcumin in Western countries to see if the dietary regimen influences curcumin potency. Nonetheless, the most convincing effects of curcumin were shown in studies conducted in Australia (167, 168).

Due to the poor bioavailability of curcumin, turmeric ingestion as powder might instinctively be thought unlikely to have therapeutic potency, particularly as the curcumin content of pure turmeric powder is only around 3% (169). However, as we have previously highlighted the neuroprotective potency of curcumin, in some studies, dietary curcuminoid consumption was positively correlated with an overall better cognitive performance in the elderly (170). In fact, this lack of bioavailability could also account for curcumin efficacy *via* its direct effects in the intestine, as it can act on intestinal barrier dysfunction as described earlier and thus tackle the subsequent systemic inflammation. Gut microbiota can also, *via* demethylation, metabolize curcumin into active metabolites, such as methexylcurcumin that has been evaluated as a potential effective synthetic analog, so the metabolization by gut microbiota into active metabolites may also exert systemic effects (171). Primarily targeting enterocytes because of this lack of bioavailability and a subsequent extensive first-pass metabolism may also account for the increased efficacy of co-administered drugs (172), supporting the use of curcumin as an add-on agent. Giving further arguments to this hypothesis, Lopresti et al. (173) have conducted a clinical trial in which the basal levels of endothelin-1 were predictive of a greater effect of curcumin administration on depressive symptoms (assessed by the inventory of depressive symptomatology). Since endothelin-1 has shown in rat models that it had a role in bacterial translocation (174), this supports the fact that ameliorating intestinal permeability and bacterial translocation may play an important role in curcumin antidepressant efficacy. The basal levels of endothelin-1 may also reflect the systemic effects of curcumin as endothelin-1 influences oxidative stress (175), HPA activity (176), and cytokine production (177).

Although curcumin has displayed an efficacy as monotherapy in placebo-controlled trials (163) or in antidepressant-controlled trials (178), this study of Lopresti et al. (173) raises the question of the subset of patients in which curcumin could be beneficial. Since curcumin has been studied in various diseases, especially inflammatory diseases, perhaps curcumin could be beneficial in patients with comorbidities. There have been two studies evaluating the use of curcumin in such conditions. The first was an 8-week randomized, double-blind, placebo-controlled trial conducted by Asadi et al. (179), testing the efficacy of curcumin on depression and anxiety in diabetic patients with polyneuropathy and showing a significant reduction of depression and anxiety in the curcumin group. The second was a 4-week randomized, double-blind, placebo-controlled trial (180) testing the antidepressant effects of curcumin in patients with systemic lupus erythematosus and that yielded positive results. There was also a positive correlation between TNF-α levels and depressive symptoms (assessed with the Beck depression inventory) that both decreased with curcumin administration. By analogy, in a meta-analysis on patients with chronic inflammatory conditions, anti-cytokine agents exhibited an antidepressant effect, especially on severely depressed patients, that was not associated with improvement in primary physical illness (181). This highlights the fact that assessing the inflammatory status of patients may be of relevance when treating patients with curcumin since immuno-inflammatory dysregulation may account for the increased efficacy of curcumin in some patients due to its anti-inflammatory properties. This hypothesis is supported by a clinical trial in which curcumin showed a greater efficacy in patients displaying “atypical” depressive symptoms (168). These symptoms are defined as leaden palsy, hypersomnia, and increased appetite, symptoms that are associated with higher levels of CRP (45). In sum, although further trials are needed, curcumin could be used in a personalized fashion in which using markers such as high-sensitive CRP could serve a biomarker-based personalized antidepressant treatment selection based on patient inflammatory status before treatment, as it has been described as a predictive factor of efficacy of certain treatments (50, 51, 182).

## Conclusion and Perspectives

**Figure 1:**
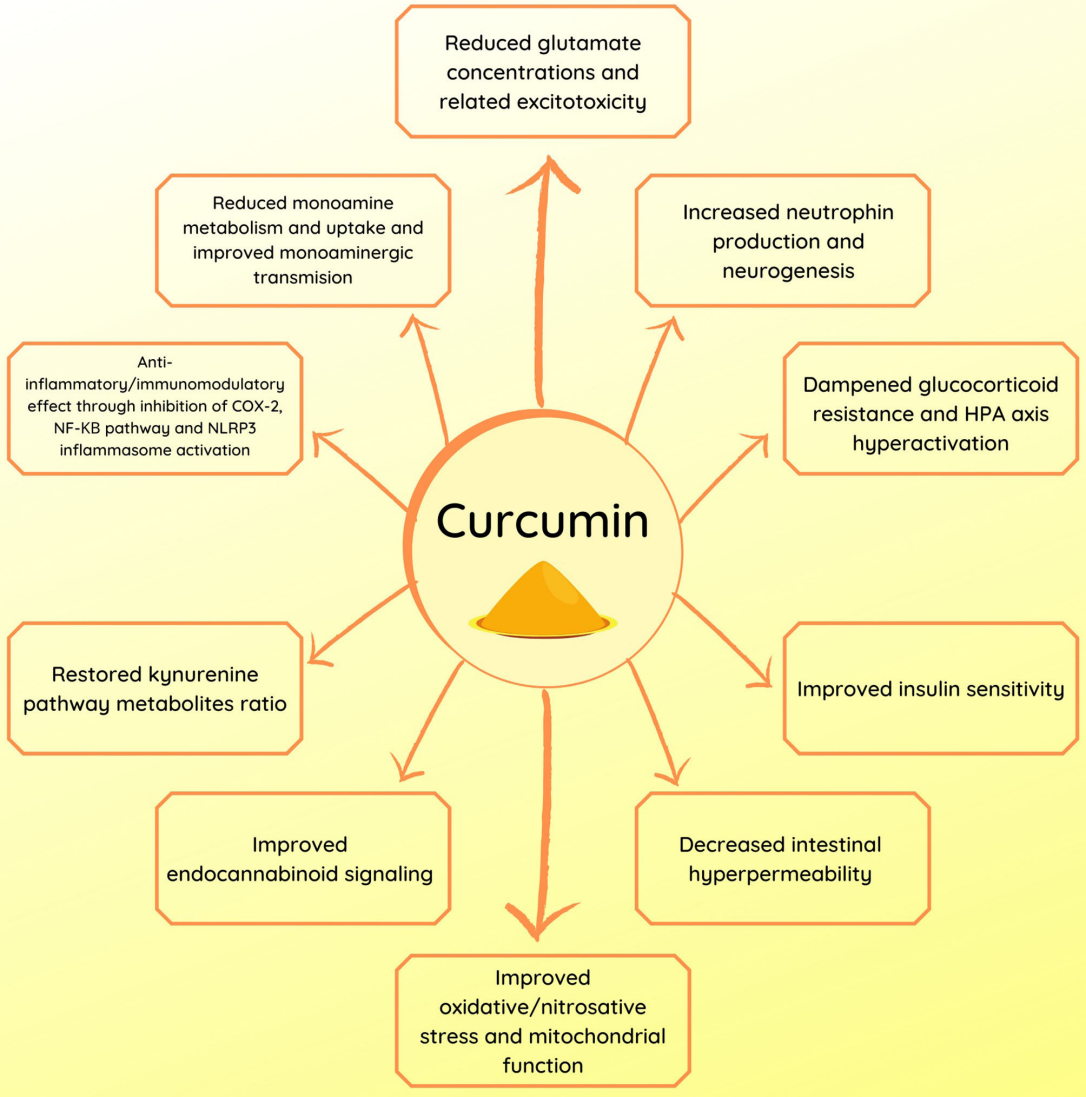
Summary of the potential mechanisms of action of curcumin in depression.

**Figure 2:**
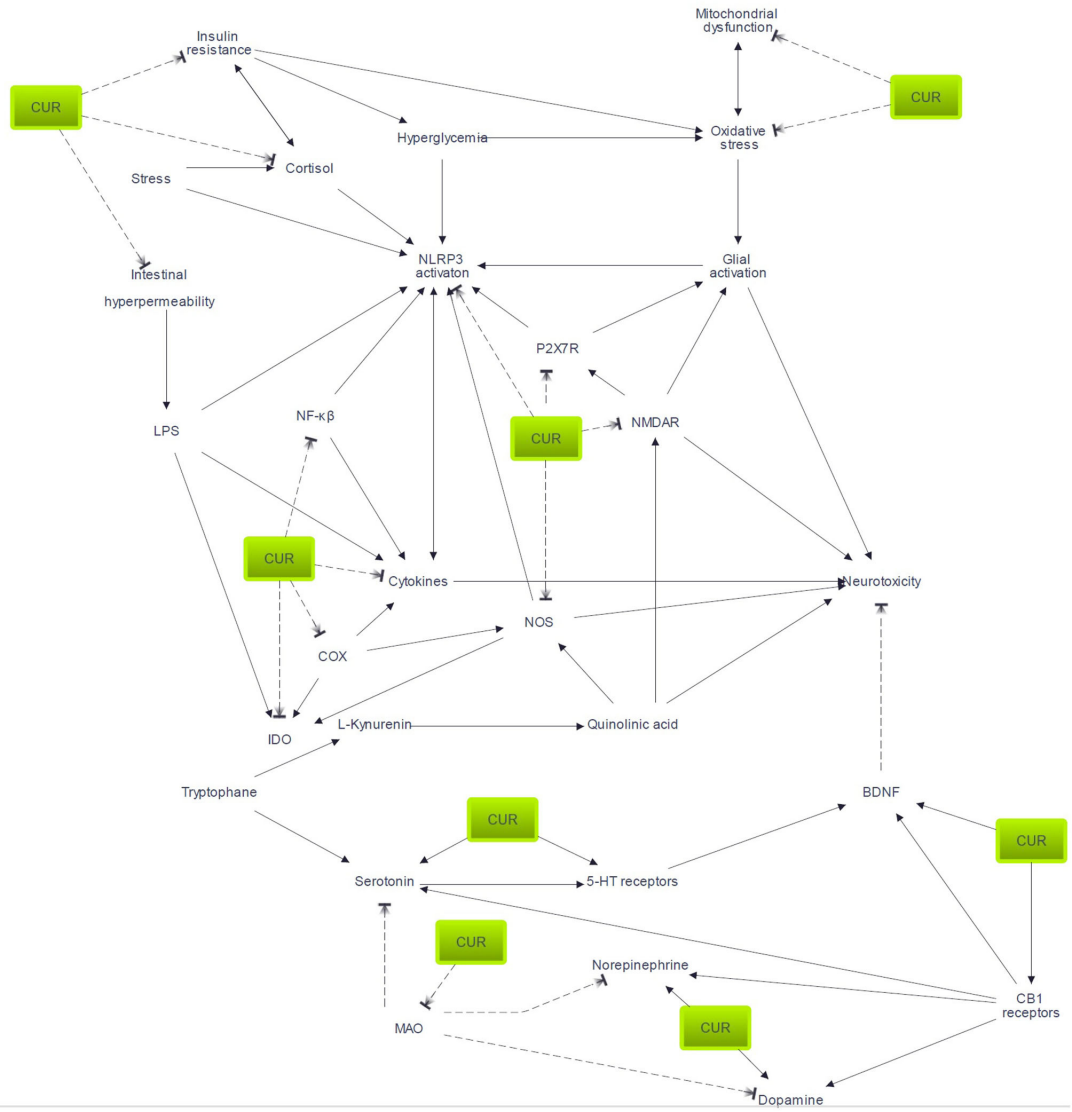
Pathways potentially involved in curcumin efficacy. Arrow indicates activation or enhancement; broken line indicates inhibitory action. 5-HT, serotonin; BDNF, brain-derived neurotrophic factor; CUR, curcumin; COX, cyclo-oxygenase; IDO, indoleamine 2,3-dioxygenase; LPS, lipopolysaccharide; MAO, monoamine oxidase; NMDAR, N-methyl-D-aspartate receptors; P2X7R, P2X7 receptor.

In this review, we highlighted the wide range of targets and modes of action of curcumin. However, as many as they are (as summarized in Figure 1), they are also interconnected (as shown in Figure 2). This illustrates the complexity of depression pathophysiology that could be described as psychophysiopathologic processes. Given the diversity of pathways involved in depression, the unidimensional nature of existing pharmacologic therapeutics may be a cause for their limited efficacy. As a consequence, an enhancement of treatment efficacy is likely to occur from therapies that target multiple mechanisms. It is under this foregoing logic that curcumin may find a place as an augmentation treatment through diverse mechanisms that have been described in this review.

Given its major anti-inflammatory properties, curcumin could also be of use in a subset of patients. As inflammatory mechanisms are relevant to some patients, those differences could account for the lack of efficacy of classical antidepressants (181).

As we have discussed in the introduction, as society evolves, patients' demands do, too. As some patients are reluctant to take medication in fear of adverse effects, alternative medicine appears to be a seductive option. Thus, curcumin could embody the dawn of nutriceuticals as anti-inflammatory and antioxidant components appear to be a possible alternative in the treatment of depression. As curcumin displays neuroprotective effects, especially against stress-induced toxicity, it also suggests the use of such molecules as prophylactic agents.

All things considered, we have highlighted that curcumin is a promising molecule as it appears to be safe and displays positive results in studies, although more clinical trials on the antidepressant effect of curcumin are still required to determine its efficacy and optimal dosage.

## Author Contributions

TR: original idea, literature review, and redaction. FB: supervision. All authors contributed to the article and approved the submitted version.

## Conflict of Interest

The authors declare that the research was conducted in the absence of any commercial or financial relationships that could be construed as a potential conflict of interest.
